# Stool Xpert^®^ MTB/RIF Ultra for TB diagnosis in children: experience from a national scale-up programme

**DOI:** 10.5588/ijtldopen.24.0334

**Published:** 2024-10-01

**Authors:** S. Kabir, S. Choudhury, T. Rahman, S.M.M. Rahman, M.K.M. Uddin, A. Nashra, A. Hossain, S. Naher, K.M.S. Towhid, L. Shahrin, S. Ahmed, P. Daru, J. Hoffmann, S. Banu

**Affiliations:** ^1^ Progamme on Emerging Infections, Infectious Diseases Division, International Centre for Diarrhoeal Disease Research, Bangladesh (icddr,b), Dhaka, Bangladesh;; ^2^ National Tuberculosis Control Programme, Dhaka, Bangladesh;; ^3^ Clinical and Diagnostic Services, Office of Executive Director, icddr,b, Dhaka, Bangladesh;; ^4^ Nutrition Research Division, icddr,b, Dhaka, Bangladesh;; ^5^ Fondation Mérieux, Lyon, France.

**Keywords:** tuberculosis, Xpert MTB/RIF Ultra, non-invasive, high TB burden country, diagnosis, Bangladesh, paediatric TB

## Abstract

**INTRODUCTION:**

We describe the programmatic scale-up of stool testing using Xpert^®^ MTB/RIF Ultra (Ultra), as recommended by the WHO to improve childhood pulmonary TB (PTB) diagnosis.

**METHODS:**

USAID's Alliance for Combating TB in Bangladesh Activity, in collaboration with the National TB Control Programme, is implementing a stool-based diagnostic approach at 51 healthcare facilities in Bangladesh to improve PTB detection. Specimens from children (<15 years) with presumptive TB were tested using ‘stool Ultra’ with routine TB diagnostics. Physicians confirmed TB diagnosis and provided treatment as per national guidelines.

**RESULTS:**

Between March 2022 and December 2023, 16,429 specimens were tested, 871 (5.3%) were positive, and 642 (73.7%) showed ‘trace detected’ results. Positivity was significantly higher among females, and children presented with ‘only cough ≥2 weeks’, ‘cough ≥2 weeks + weight loss’, or ‘fever ≥2 weeks, weight loss, fatigue + contact history’. Positivity was higher among ‘10–14 years old’ children; however, ‘trace detected’ was highest among ‘5–9 years’, followed by children aged ‘>2–<5 years’ and ‘0–2 years’.

**CONCLUSIONS:**

Testing stools using Ultra provides a more effective way of diagnosing bacteriologically positive PTB in children. However, positivity varies with presenting symptoms/criteria, highlighting the need for careful diagnostic evaluation to ensure optimum use of limited diagnostic resources.

## RÉSUMÉ

**INTRODUCTION:**

Nous décrivons l'extension programmatique du dépistage des selles à l'aide de Xpert^®^ MTB/RIF Ultra (Ultra), comme recommandé par l'OMS pour améliorer le diagnostic de la TB pulmonaire infantile (TBP).

**MÉTHODES:**

L'Alliance de l'USAID pour la lutte contre la TB au Bangladesh L'activité, en collaboration avec le Programme national de lutte contre la TB, met en œuvre une approche de diagnostic basée sur les selles dans 51 établissements de santé au Bangladesh afin d'améliorer la détection du TBP. Des échantillons prélevés chez des enfants (<15 ans) atteints de TB présumée ont été analysés à l'aide de « selles Ultra » avec un diagnostic de routine de la tuberculose. Les médecins ont confirmé le diagnostic de TB et fourni un traitement conformément aux directives nationales.

**RÉSULTATS:**

Entre mars 2022 et décembre 2023, 16 429 échantillons ont été testés, 871 (5,3%) étaient positifs et 642 (73,7%) présentaient des résultats de « traces détectées ». La positivité était significativement plus élevée chez les femmes et les enfants présentaient « seulement de la toux ≥2 semaines », « de la toux ≥2 semaines + perte de poids » ou « de la fièvre ≥2 semaines, perte de poids, fatigue + antécédents de contact ». La positivité était plus élevée chez les enfants de 10 à 14 ans ; Cependant, les « traces détectées » étaient les plus élevées chez les enfants de 5 à 9 ans, suivis des enfants âgés de « >2 à <5 ans » et de « 0 à 2 ans ».

**CONCLUSIONS:**

L'analyse des selles à l'aide d'Ultra offre un moyen plus efficace de diagnostiquer la TBP bactériologiquement positive chez les enfants. Cependant, la positivité varie selon les symptômes/critères présentés, ce qui souligne la nécessité d'une évaluation diagnostique minutieuse pour assurer une utilisation optimale des ressources diagnostiques limitées.

TB is a leading cause of death globally. In 2022, approximately 10.6 million people were affected, of which 1.3 million (12%) were children (<15 years).^1^ Bangladesh has a high burden of TB and multidrug-resistant TB (MDR-TB), and among the 379,000 estimated people with TB (PWTB), about 30,000 (8%) are expected to be children. However, in 2022, only 4% (12,000) of the total PWTB notified to the National TB Control Programme (NTP) were children.^1^ Diagnosing TB in children remains a major challenge due to the non-specificity of TB symptoms, and up to 50% of children might be asymptomatic in the early stages of the disease.^2^ Also, chest X-ray (CXR) and tuberculin skin test (TST) are widely used investigations; however in children, CXR frequently exhibits non-specific findings and TST often shows false-negative results.^3–6^ Furthermore, children cannot always expectorate sufficient sputum,^6,7^ and collection and testing of alternative respiratory specimens (gastric lavage/induced sputum) are not always possible in resource-limited settings (due to their invasive nature and lack of trained personnel).^3,4,8,9^ The yield of culture or other molecular diagnostics is also low in respiratory specimens due to the paucibacillary nature of TB in children.^9,10^ As a consequence, physicians usually depend on clinical features and supporting investigations for diagnosis in children, which may lead to misdiagnosis and contribute to the gap between estimations and notifications.^11,12^

TB bacilli in swallowed sputum that passes through the gastrointestinal tract can be detected using molecular diagnostics of stool specimens.^10,13,14^ Previous studies have revealed stool to be a promising specimen for TB detection, especially in children,^6,8,15–18^ with Xpert^®^ MTB/RIF Ultra (Ultra; Cepheid, Sunnyvale, CA, USA) assay being more sensitive than conventional Xpert MTB/RIF (Xpert).^14,19,20^ A study from Bangladesh found bacteriological confirmation to be around two times higher for stool specimens than induced sputum (16.1% vs 6.5%) in children.^19^ The sensitivity and specificity of ‘Ultra on stool’ (stool Ultra) were respectively 58.1% and 88.1%, compared to ‘bacteriological confirmation on induced sputum’.^19^ These findings led to this strategy being incorporated into national guidelines and a National Strategic Plan (2024–2030) for Bangladesh. Currently, the NTP is conducting a countrywide scale-up of stool testing and is in the transitional phase of replacing conventional Xpert with Ultra.^21,22^ The WHO has recommended stool testing by Xpert and Ultra for childhood pulmonary TB (PTB) diagnosis; however, data is limited on the use of ‘stool Ultra’ in resource-limited, high-burden countries.^23^ Here, we describe the programmatic implementation of ‘stool Ultra’ to improve TB diagnosis in Bangladesh, an approach that other resource-limited countries might also be keen to develop.

## METHODS

Bangladesh is a lower middle-income country in South Asia, with a population of 165 million,^24^ and the country's eight administrative divisions are divided into 64 districts.^24^ In collaboration with the NTP, USAID's Alliance for Combating TB in Bangladesh Activity is implementing a stool-based TB diagnostic approach at 51 healthcare facilities across 30 districts in five divisions. Approximately 100, 120 and 150 children respectively visit each of these primary, secondary and tertiary facilities each day for healthcare services for various illnesses. The facilities are well-staffed with physicians/paediatricians and include pathology laboratories for essential testing, and TST and X-ray are available locally. Most have GeneXpert, but if not, patients are referred, or their specimens transported to nearby Xpert testing sites.

We present data from March 2022 to December 2023 from this ongoing programmatic activity. Using a cross-sectional approach, all <15-year-old children visiting facilities during this period were screened (*n* = 3,339,193) for PTB. We used a standardised toolkit for symptoms or criteria including 1) continuous cough for ≥2 weeks; 2) continuous fever for ≥2 weeks; 3) weight loss/not gaining weight for last 3 months; 4) reduced playfulness/fatigue; 5) history of contact with TB in the family within the past 12 months (contact history); 6) breathing difficulty. Children with any of these symptoms/criteria were considered to have presumptive PTB and were evaluated by local physicians/paediatricians the same day and referred for Xpert testing on sputum/induced sputum/gastric lavage (if possible), TST and CXR. Of the 83,358 children identified with presumptive PTB, 16,429 underwent ‘stool Ultra’ testing following standard procedures^25^ at the nearby Ultra-equipped International Centre for Diarrhoeal Disease Research, Bangladesh (icddr,b) run TB Screening and Treatment Centres, or transported to the icddr,b Mycobacteriology Laboratory, Dhaka, Bangladesh. Specimens were processed and tested following an optimised and validated procedure.^19^ Stool specimen collection, transportation and processing for Ultra testing are described in the accompanying Supplementary Data.

All test reports were delivered as per availability. The final PTB diagnosis was made by treating physicians based on test reports or clinical features. Physicians followed WHO's standard criteria for clinical diagnosis of TB.^21^ Children diagnosed with PTB were referred to local directly observed treatment (DOT) centres for TB treatment per national guidelines. Children not diagnosed with TB were treated by physicians accordingly.

Data were collected in structured electronic forms through a web-based Android app. Screeners collected data on socio-demography (age/sex/contact information/address) and criteria for presumptive PTB (yes/no), MT (positive/negative), CXR (TB suggestive/TB non-suggestive/normal); stool Ultra (MTB detected/trace detected/MTB not detected/invalid/error), rifampicin resistance for positive ‘stool Ultra’ result (detected/not detected/indeterminate), TB diagnosis (yes/no), anti-TB treatment initiation (yes/no). Data were regularly reviewed for quality assurance and uploaded directly to the main server. All invalid/error results were retested to get valid results.

Data were analysed using statistical software Stata/SE v17 (StataCorp, College Station, TX, USA) and relevant statistical methods. We calculated summary statistics (mean, median, standard deviation, etc.) to report sociodemographic details of children and their clinical symptoms. We performed the χ^2^ test to assess the relationship between ‘stool Ultra’ and its positivity. ‘MTB detected’ and ‘trace detected’ results were considered positive. Unadjusted logistic regression was conducted to measure the association of ‘stool Ultra’ positivity with children's sociodemographic and symptoms/criteria. Multivariate logistic regression was then performed to adjust significant covariates, e.g. ‘age’, ‘sex’, ‘cough ≥2 weeks’, ‘fever ≥2 weeks’, ‘weight loss’, ‘breathing difficulty’, ‘contact history’, ‘only cough ≥2 weeks’, ‘cough ≥2 weeks and weight loss’ and ‘fever ≥2 weeks, weight loss, fatigue + contact history’ to determine factors related to test positivity by ‘stool Ultra’. We also developed ‘Model 1’ and ‘Model 2’, considering under-5 children and older, respectively, following the same criteria. Odds ratios (ORs) with their 95% confidence intervals (CIs) were measured considering two-tailed tests of significance. Statistical significance was determined at *P* <0.05 with 95% CIs.

The protocol was approved by icddr,b's Institutional Review Board, constituting the Research Review Committee and Ethical Review Committee. We initiated this programmatic intervention after securing permission from local health authorities and facilities with the NTP's support letter. This was part of an existing healthcare system, and no one had undergone additional procedures for this activity. Project staff aided physicians in early TB detection through history taking, where the purpose was explained to respondents, and their verbal consent was secured. We strictly maintained the confidentiality of children and their health status and ensured data anonymity. Only concerned health personnel, implementers and respondents/participants can access anonymous data.

## RESULTS

The mean age of children with presumptive PTB tested using ‘stool Ultra’ was 5.5 (standard deviation [SD] ±3.8) years. From a total of 16,429 with presumptive PTB, 9,644 (58.7%) were male, 3,900 (23.7%) were ‘0–2 years old’, and 5,163 (31.4%) were ‘5–9 years old’. A total of 13,291 (80.9%) had cough for ≥2 weeks, 13,225 (80.5%) had a fever for ≥2 weeks, 12,612 (76.8%) had a history of weight loss, 3,372 (20.5%) were fatigue, and 2,428 (14.8%) had contact history (Table 1).

**Table 1.** Characteristics of children with presumptive PTB tested with ‘stool Ultra’ and ‘positive’ under programmatic activity in Bangladesh.

Characteristics	Presumptive PTB tested with ‘stool Ultra’ *n* (%)*	Test results on ‘stool Ultra’			*P*-value^§^
		MTB detected *n* (%)^†^	Trace detected *n* (%)^†^	Total ‘positive’ *n* (%)^‡^	
Total children	16,429	229 (26.3)	642 (73.7)	871 (5.3)	
Age, years					
Mean ± SD	5.5 ± 3.8	6.8 ± 4.9	5.5 ± 3.8	5.8 ± 4.1	
≤2	3,900 (23.7)	61 (28.0)	157 (72.0)	218 (5.6)	
>2–<5	3,546 (21.6)	28 (18.1)	127 (81.9)	155 (4.4)	
5–9	5,163 (31.4)	43 (16.7)	215 (83.3)	258 (5.0)	
10–14	3,820 (23.3)	97 (40.4)	143 (59.6)	240 (6.3)	
Sex					
Male	9,644 (58.7)	102 (21.7)	369 (78.3)	471 (4.9)	0.004^¶^
Female	6,785 (41.3)	127 (31.8)	273 (68.3)	400 (5.9)	0.004^¶^
TB-suggestive symptoms					
Cough ≥2 weeks	13,291 (80.9)	185 (26.7)	507 (73.3)	692 (5.2)	0.261
Fever ≥2 weeks	13,225 (80.5)	200 (27.0)	540 (73.0)	740 (5.6)	0.001^¶^
Weight loss	12,612 (76.8)	190 (27.2)	508 (72.8)	698 (5.5)	0.015^¶^
Fatigue	3,372 (20.5)	54 (28.6)	135 (71.4)	189 (5.6)	0.376
Breathing difficulty	1,787 (10.9)	25 (21.2)	93 (78.8)	118 (6.6)	0.009^¶^
Contact history	2,428 (14.8)	55 (35.0)	102 (65.0)	157 (6.5)	0.005^¶^
TB diagnosis by physician		224 (28.6)	559 (71.4)	783 (89.9)^†^	<0.01^¶^

* Column percentage.

† Percentage among resulted positive on 'stool Ultra'.

‡ Percentage among tested for 'stool Ultra'.

§ *P* value compared to tested positive and negative.

¶ Significant if *P* < 0.05.

PTB = pulmonary TB; MTB = *Mycobacterium tuberculosis*; SD = standard deviation.

### ‘Stool Ultra’ positivity and details of 'MTB detected' and 'trace detected' results

A total of 871 (5.3%) children tested ‘positive’ by ‘stool Ultra’, 642 (73.7%) were ‘trace detected’, and eight (0.9%) were rifampicin-resistant. Among those tested, 218 (5.6%) were ‘0–2 years old’, 258 (5.0%) were ‘5–9 years old’, and 240 (6.3%) were ‘10–14 years old’ (Table 1). Segregating ‘positive’ results into two categories, ‘MTB detected’ was found in 28.0% (61/218) of ‘0–2 years old’ and 40.4% (97/240) of ‘10–14 years old’. ‘Trace detected’ was found in 81.9% (127/155) of ‘>2–<5 years old’ and 83.3% (215/255) of ‘5–9 years old’. A total of 783 (89.9%) children with ‘positive’ results received TB treatment (Table 1).

### ‘Stool Ultra’ positivity and different symptoms/criteria

‘Stool Ultra’ positivity was significantly higher among children with ‘only cough for ≥2 weeks’ (14, 2.8%) than children with another single symptom/criterion. For combinations of two TB-suggestive symptoms/criteria, positivity was significantly higher among children presenting with ‘cough for ≥2 weeks + fever for ≥2 weeks’ (*n* = 76, 4.7%) and ‘cough for ≥2 weeks + weight loss’ (*n* = 32, 3.8%). For three or more symptoms/criteria, children with ‘fatigue + breathing difficulty + contact history’ (*n* = 1, 25%), ‘fever ≥2 weeks + weight loss + fatigue + contact history’ (*n* = 7, 14.9%), and ‘weight loss + fatigue + breathing difficulty + contact history’ (*n* = 1, 33.3%) had significantly higher test positivity. Another combination of symptoms/criteria, ‘cough ≥2 weeks + fever ≥2 weeks + weight loss’, had 28% (*n* = 244) test positivity; however, this was not statistically significant. The distribution of symptoms/criteria among children tested with ‘stool Ultra’ and total and age-segregated children with ‘positive’ results is shown in Table 2.

**Table 2.** Distribution of symptoms/criteria suggestive of TB among children with presumptive PTB and tested ‘positive’ using ‘stool Ultra’ among under 5-year olds and 5-14-year olds.

TB-suggestive symptoms	Children with presumptive PTB (*n* = 16,429) *n* (%)*	Under-5 years old 'positive' on 'stool Ultra' (*n* = 373) *n* (%)^†^	5–14 years old 'positive' on 'stool Ultra' (*n* = 498) *n* (%)^†^	'Children positive' on 'stool Ultra' (*n* = 871) *n* (%)^†^	*P*-value^‡^
Only cough ≥2 weeks	497 (3.0)	8 (2.1)	6 (1.2)	14 (2.8)	0.012^§^
Only fever ≥2 weeks	134 (0.8)	3 (0.8)	2 (0.4)	5 (3.7)	0.416
Only weight loss	87 (0.5)	3 (0.8)	2 (0.4)	5 (5.7)	0.852
Only fatigue	4 (0.02)	0 (0.0)	1 (0.2)	1 (25.0)	0.079
Only breathing difficulty	9 (0.1)	0 (0.0)	0 (0.0)	0 (0)	0.478
Only contact history	86 (0.5)	1 (0.3)	0 (0.0)	1 (1.2)	0.086
Cough ≥2 weeks + fever ≥2 weeks	1,633 (9.9)	35 (9.4)	41 (8.2)	76 (4.7)	0.215
Cough ≥2 weeks + weight loss	841 (5.1)	14 (3.8)	18 (3.6)	32 (3.8)	0.047^§^
Cough ≥2 weeks + fatigue	66 (0.4)	1 (0.3)	1 (0.2)	2 (3)	0.409
Cough ≥2 weeks + breathing difficulty	120 (0.7)	5 (1.3)	2 (0.4)	7 (5.8)	0.794
Cough ≥2 weeks + contact history	114 (0.7)	6 (1.6)	2 (0.4)	8 (7)	0.412
Fever ≥2 weeks + weight loss	1,018 (6.2)	22 (5.9)	33 (6.6)	55 (5.4)	0.880
Fever ≥2 weeks + fatigue	44 (0.3)	1 (0.3)	2 (0.4)	3 (6.8)	0.653
Fever ≥2 weeks + breathing difficulty	22 (0.1)	2 (0.5)	0 (0.0)	2 (9.1)	0.427
Fever ≥2 weeks + contact history	63 (0.4)	1 (0.3)	1 (0.2)	2 (3.2)	0.451
Weight loss + fatigue	93 (0.6)	1 (0.3)	3 (0.6)	4 (4.3)	0.666
Weight loss+ breathing difficulty	19 (0.1)	0 (0.0)	0 (0.0)	0 (0.0)	0.302
Weight loss + contact history	91 (0.6)	0 (0.0)	6 (1.2)	6 (6.6)	0.581
Fatigue + breathing difficulty	6 (0.04)	0 (0.0)	0 (0.0)	0 (0.0)	0.562
Fatigue + contact history	5 (0.03)	0 (0.0)	0 (0.0)	0 (0.0)	0.597
Breathing difficulty + contact history	4 (0.02)	0 (0.0)	1 (0.2)	1 (25)	0.079
Cough ≥2 weeks + fever ≥2 weeks + weight loss	4,699 (28.6)	93 (24.9)	151 (30.3)	244 (5.2)	0.691
Fever ≥2 weeks + weight loss + fatigue	359 (2.2)	9 (2.4)	16 (3.2)	25 (7)	0.155
Weight loss + fatigue+ breathing difficulty	16 (0.1)	0 (0.0)	0 (0.0)	0 (0)	0.344
Fatigue + breathing difficulty + contact history	1 (0.01)	1 (0.3)	0 (0.0)	1 (100)	<0.01^§^
Fever ≥2 weeks + weight loss + fatigue + breathing difficulty	24 (0.2)	0 (0.0)	3 (0.6)	3 (12.5)	0.115
Fever ≥2 weeks + weight loss + fatigue + contact history	47 (0.3)	2 (0.5)	5 (1.0)	7 (14.9)	0.003^§^
Weight loss + fatigue + breathing difficulty + contact history	3 (0.0)	0 (0.0)	1 (0.2)	1 (33.3)	0.030^§^

* Percentage among children with presumptive PTB tested for 'stool Ultra'.

† Percentage among positive on 'stool Ultra' in a particular age group.

‡ χ^2^ was calculated between 'tested positive' and 'tested negative' among TB-suggestive symptoms/criteria.

§ Significant if *P* < 0.05.

PTB = pulmonary TB.

### Factors associated with ‘stool Ultra’ positivity

For univariate and multivariate analyses, females had 22% higher odds of ‘stool Ultra’ positivity than males (adjusted OR [aOR] 1.26, 95% CI 1.06–1.49). TB-suggestive symptoms/criteria, including fever ≥2 weeks, weight loss, breathing difficulty, and contact history, were significantly associated with higher odds of ‘stool Ultra’ positivity in univariate and multivariate analyses. In multivariate analysis, after adjusting covariates, the presence of specific combinations of symptoms/criteria as ‘fever ≥2 weeks + weight loss + fatigue + contact history’ showed varying degrees of association with ‘stool Ultra’ positivity (aOR 2.28, 95% CI 0.99–5.26; *P* = 0.053) (Table 3).

**Table 3.** Univariate and multivariate logistic regression analyses of demographics and symptoms/criteria associated with 'stool Ultra' positivity among children with presumptive PTB.

Covariates	cOR (95% CI)	*P*-value	aOR (95% CI)	*P*-value
Age: 5–14 years (reference: under-5)	1.10 (0.97–1.28)	0.129	1.10 (0.96–1.27)	0.174
Sex: female (reference: male)	1.22 (1.06–1.4)	0.005^*^	1.22 (1.06–1.4)	0.005*
Cough ≥2 weeks (reference: no)	0.91 (0.77–1.07)	0.262	0.93 (0.78–1.11)	0.443
Fever ≥2 weeks (reference: no)	1.39 (1.15–1.68)	0.001^†^	1.33 (1.05–1.69)	0.020*
Weight loss (reference: no)	1.23 (1.04–1.46)	0.015^*^	1.16 (0.97–1.39)	0.101
Fatigue (reference: no)	1.08 (0.91–1.27)	0.376		
Breathing difficulty (reference: no)	1.3 (1.07–1.59)	0.009^*^	1.35 (1.1–1.66)	0.004^*^
Contact history (reference: no)	1.29 (1.08–1.54)	0.006^*^	1.25 (1.04–1.51)	0.017^*^
TB-suggestive symptoms or criteria				
Only cough ≥2 weeks (reference: no)	0.51 (0.3–0.87)	0.014^*^	0.8 (0.44–1.46)	0.476
Only fever ≥2 weeks (reference: no)	0.69 (0.28–1.69)	0.418		
Only weight loss (reference: no)	1.09 (0.44–2.7)	0.852		
Only fatigue (reference: no)	5.96 (0.62–57.37)	0.122		
Only contact history (reference: no)	0.21 (0.03–1.5)	0.12		
Cough ≥2 weeks + fever ≥2 weeks (reference: no)	0.86 (0.67–1.09)	0.215		
Cough ≥2 weeks + weight loss (reference: no)	0.7 (0.49–1)	0.048^*^	0.95 (0.61–1.46)	0.81
Cough ≥2 weeks + fatigue (reference: no)	0.56 (0.14–2.28)	0.416		
Cough ≥2 weeks + breathing difficulty (reference: no)	1.11 (0.51–2.38)	0.794		
Cough ≥2 weeks + contact history (reference: no)	1.35 (0.66–2.78)	0.413		
Fever ≥2 weeks + weight loss (reference: no)	1.02 (0.77–1.35)	0.88		
Fever ≥2 weeks + fatigue (reference: no)	1.31 (0.4–4.23)	0.654		
Fever ≥2 weeks + breathing difficulty (reference: no)	1.79 (0.42–7.66)	0.434		
Fever ≥2 weeks + contact history (reference: no)	0.58 (0.14–2.4)	0.456		
Weight loss + fatigue (reference: no)	0.8 (0.29–2.19)	0.667		
Weight loss + contact history (reference: no)	1.26 (0.55–2.9)	0.582		
Breathing difficulty + contact history (reference: no)	5.96 (0.62–57.37)	0.122		
Cough ≥2 weeks + fever ≥2 weeks + weight loss (reference: no)	0.97 (0.83–1.13)	0.691		
Fever ≥2 weeks + weight loss + fatigue (reference: no)	1.35 (0.89–2.03)	0.156		
Fever ≥2 weeks + weight loss + fatigue + breathing difficulty (reference: no)	2.56 (0.76–8.59)	0.129		
Fever ≥2 weeks + weight loss + fatigue + contact history (reference: no)	3.14 (1.4–7.04)	0.005^*^	2.28 (0.99–5.26)	0.053
Weight loss + fatigue + breathing difficulty + contact history (reference: no)	8.94 (0.81–98.72)	0.074		

* Significance set at *P* <0.05.

PTB = pulmonary TB; cOR = crude odds ratio; CI = confidence interval; aOR = adjusted OR.

While segregating age groups, after adjusting covariates, Model 1 showed ‘under 5’ children having ‘fever ≥2 weeks’, ‘contact history’ and ‘fever ≥2 weeks + breathing difficulty’ (aOR 5.49, 95% CI 1.14–26.58; *P* = 0.034) were significantly associated with higher odds of ‘stool Ultra’ positivity. In Model 2, among ‘5–14 years old’ children, ‘female’, ‘fever ≥2 weeks’, ‘breathing difficulty’, ‘contact history’, ‘fever ≥2 weeks + weight loss + fatigue + contact history’ (aOR 3.05, 95% CI 1.17–7.98; *P* = 0.023) were significantly associated with a higher likelihood of ‘stool Ultra’ positivity (see Supplementary Table S1).

## DISCUSSION

We have assessed the performance of ‘stool Ultra’ testing under programmatic conditions for PTB diagnosis in children. Our key observations are 1) ‘stool Ultra’ positivity was 5.3% with 73.7% ‘trace detected’; 2) positivity was higher among ‘10–14 years old’ children and ‘trace detected’ was highest among ‘5–9 years old’ children, followed by ‘>2–<5 years old’ and ‘0–2 years old’ groups; 3) females were associated with higher odds of ‘stool Ultra’ positivity compared to males; 4) ‘Stool Ultra’ positivity was significantly higher among children presented with single symptom/criterion like ‘fever ≥2 weeks’, ‘breathing difficulty’, ‘contact history’, ‘only cough ≥2 weeks’, or combination of symptoms/criteria like ‘cough ≥2 weeks’ + ‘weight loss’; positivity was also higher among children presented with ‘fever ≥2 weeks + weight loss + fatigue + contact history’ among non-coughed children (*P* = 0.053).

We observed ‘stool Ultra’ positivity of 5.3% among tested children, suggesting that one in every 20 children with presumptive PTB is expected to be ‘positive’. This is consistent with findings from Nigeria and Vietnam.^26,27^ In contrast, positivity was higher (13.4%) in a previous study in Bangladesh,^19^ which may be due to differences in the study population (the earlier study was on children admitted to hospital, who might have had highly TB-suggestive criteria). Another study in Tajikistan showed 2.9% positivity of ‘stool Ultra’, which is lower than our finding and might be due to differences in specimen quantity and centrifugation (we used a larger volume and centrifugation) before testing.^28^ Among ‘positive’, 73.7% were ‘trace detected’, similar to the previous study in Bangladesh.^19^

Test positivity was higher among ‘10–14 years old’ children, which might be due to a higher bacillary load in the stools of older children and is consistent with a previous study.^9^ Conversely, our ‘trace detected’ result was highest in younger groups (‘5–9 years old’, followed by ‘>2–<5 years’ and ‘0–2 years old’). We were unable to find literature relevant to this finding, but we hypothesise that this could be due to the paucibacillary nature of TB in children, especially in younger age groups. As Ultra can detect *Mycobacterium tuberculosis* complex from specimens with low bacillary loads (minimal load is categorised by ‘trace detected’), younger children can show ‘trace detected’ results.^29^ Therefore, stool testing by Ultra might be helpful for younger children as bacteriological confirmation is challenging compared to older children who can expectorate sputum (either spontaneously or with assistance) for microbiological testing and confirmation.^30^

Another important observation was the higher number of females ‘positive’ on ‘stool Ultra’ compared to males (*P* < 0.001), specifically among older (10–14 years old) children (Model-2). This was consistent with several other studies where a significantly higher number of female children were diagnosed with TB.^31–33^ A possible explanation might be the poor nutritional status of female children, which increases their risk of developing TB.^33,34^ Other studies have hypothesised that female children with TB-affected mothers are at greater risk of being infected by the mother.^32,33^ However, this observation requires further research in high TB burden settings.

‘Stool Ultra’ positivity was significantly higher among children who presented with ‘only cough ≥2 weeks’ without other symptoms/criteria. Test positivity was also higher for a combination of symptoms/criteria: ‘cough ≥2 weeks + weight loss’, and in children with no cough, ‘fever ≥2 weeks + weight loss + fatigue + contact history’ (*P* = 0.053). This last combination of symptoms/criteria correlates with a positive test and we recommend ‘stool Ultra’ testing for TB in children. Considering the age segregations, significant factors associated with higher odds of ‘stool Ultra’ positivity in ‘Model 1’ were ‘fever ≥2 weeks’, ‘contact history’ and ‘fever ≥2 weeks + breathing difficulty’. Similarly, for ‘Model 2’, significant associated factors were ‘fever ≥2 weeks’, ‘breathing difficulty’, ‘contact history’, and ‘fever ≥2 weeks + weight loss + fatigue + contact history’. We were unable to find studies supporting these age-specific findings. Nevertheless, a prospective, community-based study showed that the combined presence of three well-defined symptoms (persistent, non-remitting cough >2 weeks and weight loss during the preceding three months) provided good diagnostic accuracy for TB in non-HIV children.^35^

A limitation of this analysis is that it was performed on data from programmatic activities. Hence, the selection criteria for stool testing might vary according to physicians' opinions across sites.

## CONCLUSION

Testing using ‘stool Ultra’ provides an effective means of diagnosing children with bacteriologically positive TB. However, positivity varies according to the presenting symptoms, suggesting there is a need for a careful diagnostic evaluation to ensure the optimum use of limited diagnostic resources.

## Supplementary Material


