# Supplementary material for: Stool Xpert® MTB/RIF Ultra for TB diagnosis in children: experience from a national scale-up programme
Source: IJTLD Open. 2024 Oct 1;1(10):437–42. doi: 10.5588/ijtldopen.24.0334 (PMC11467856; doi:10.5588/ijtldopen.24.0334)
Supplement: Supplementary file 1 [file ijtldopen24-0334_SupplementaryData1.docx]

Supplementary Data File:

1. Details related to stool specimen collection, transportation, processing and testing by Xpert MTB/RIF Ultra assay
2. **Table S1:** Univariate and multivariate logistic regression analyses of demographics, symptoms or criteria associated with 'stool Ultra' positivity

1. Details related to stool specimen collection, transportation, processing and testing by Xpert MTB/RIF Ultra assay

Stool specimen collection

When a presumptive Ch-PTB was advised for stool Xpert MTB/RIF Ultra (Ultra) assay testing, the purpose of collection of stool specimen was well explained to the parent or guardian. A sterile container and a piece of foil paper were provided to the parent/guardian of the presumptive Ch-PTB patient for collecting stool specimen. Parent/guardian were asked to store a portion of early morning stool specimen in the container using the spoon provided inside. The parent/guardian were advised to fill two-third (no less than half) of the container with the stool specimen. After collection, the parents were asked transport it to respective healthcare facilities as early as possible.

Transportation of stool specimen

After receiving the stool container, the field staff checked and sealed the container cap properly with parafilm. The sealed container was labelled properly and kept in a Ziploc bag. The staff then stored the Ziploc bag containing the stool container in a cool box containing icepack. A temperature monitor was placed on the container to record and ensure maintenance of temperature. The cool box containing the stool container was transported from the field sites to icddr,b Mycobacteriology Laboratory maintaining a temperature of 2°C - 8°C.

Stool specimen processing

An optimized method was developed for stool specimen processing. Approximately, 2 gm of stool specimen was collected into a 50 ml centrifuge tube from each children with presumptive PTB. Equal volume of sterile normal saline (0.9% NaCl) was added to the stool specimen and mixed well by vortexing. Then normal saline was added in the centrifuge tube up to 30 ml mark and incubated at room temperature for 30 minutes. After incubation, 10 ml of supernatant from the tube was transferred into a new 50 ml centrifuge tube which was then decontaminated and

concentrated following the Petroff's NaOH method (1). Equal volume of NALC-NaOH-Na-citrate solution (0.5% N-acetyl-L-citrate, 4% NaOH, and 2.94% Na-citrate) was added into this centrifuge tube and incubated for 20 minutes at room temperature. The tube was then filled with sterile phosphate buffer saline (PBS) (pH 6.8) up to 40 ml mark and centrifuged twice at 3000g for 20 minutes. After centrifugation, the supernatant was discarded and the resulting sediment was saved for Ultra testing.

Stool testing by Ultra assays

A portion of processed stool was tested with Ultra according to the manufacturer's instructions (2). The test integrates specimen processing and PCR in a disposable plastic cartridge containing all reagents required for bacterial lysis, nucleic acid extraction, amplification, and amplicon detection. Specimen reagent buffer was added to the aliquoted specimen in a 2:1 ratio. The closed container containing specimen and buffer mixture were manually agitated twice during a 15 minutes of incubation period at room temperature. Then 2 ml of the inactivated mixture were transferred to the Ultra test cartridges. Cartridges were inserted into the test platform and the automatically generated results were recorded. According to WHO Technical Expert Consultation findings on Ultra assay, 'trace call' should be considered to be true positive results for use in clinical decisions in children (3).

Interpretation of Ultra result

- 'MTB Detected'- Positive results for presence of *Mycobacterium tuberculosis* complex in stool. Results of 'MTB Detected' results may show several burdens of bacilli such as high, medium, low, very low and trace
- 'MTB Not-detected'- Negative Ultra results in stool
- 'Invalid' or 'Error'- In case of 'Invalid' or 'Error' results, tests are repeated to obtain a valid result. Some of the reasons for 'Invalid' or 'Error' results are due to technical problems, inappropriate specimen quantity, incorrect temperature, dirt on filter cartridge quality, module problem etc. However, all invalid/error results are retested and valid results are provided
- 'RIF-Resistance'- The result will be given either as "MTB detected, rifampicin resistance detected" or "MTB detected, rifampicin resistance not detected (or indeterminate)"

Table S1. Univariate and multivariate logistic regression analyses of demographics, symptoms or criteria associated with 'stool Ultra' positivity**.**

	Model 1: Children aged <5 years				Model 2: Children aged 5–14 years			
Covariates	cOR (95% CI)	*P*-value	aOR (95% CI)	*P*-value	cOR (95% CI)	*P*-value	aOR (95% CI)	*P*-value
Sex: female (reference: male)	1.06 (0.86–1.31)	0.598			1.35 (1.13–1.62)	0.001*	1.36 (1.14–1.63)	0.001*
Cough ≥2 weeks (reference: no)	0.78 (0.6–1.02)	0.066			1.02 (0.82–1.27)	0.886		
Fever ≥2 weeks (reference: no)	1.37 (1.04–1.82)	0.025*	1.39 (1.05–1.84)	0.02*	1.39 (1.07–1.8)	0.013*	1.39 (1.07–1.81)	0.013*
Weight loss (reference: no)	1.18 (0.92–1.5)	0.186			1.27 (1–1.62)	0.053		
Fatigue (reference: no)	1.12 (0.87–1.45)	0.381			1.04 (0.84–1.3)	0.709		
Breathing difficulty (reference: no)	1.24 (0.94–1.65)	0.131			1.42 (1.07–1.89)	0.016*	1.46 (1.1–1.95)	0.009*
Contact history (reference: no)	1.48 (1.14–1.92)	0.003*	1.51 (1.16–1.96)	0.002*	1.15 (0.9–1.47)	0.269		
TB-suggestive symptoms								
Only cough ≥2 weeks (reference: no)	0.55 (0.27–1.12)	0.1			0.48 (0.21–1.08)	0.075		
Only fever ≥2 weeks (reference: no)	0.89 (0.28–2.84)	0.841			0.52 (0.13–2.14)	0.367		
Only weight loss (reference: no)	1.59 (0.49–5.17)	0.445			0.74 (0.18–3.06)	0.677		
Cough ≥2 weeks + fever ≥2 weeks (reference: no)	0.8 (0.56–1.14)	0.218			0.93 (0.67–1.29)	0.657		

Cough ≥2 weeks + weight loss(reference: no)	0.68 (0.39–1.17)	0.164			0.71 (0.44–1.15)	0.165		
Cough ≥2 weeks + fatigue (reference: no)	0.68 (0.09–4.99)	0.701			0.47 (0.06–3.45)	0.46		
Cough ≥2 weeks + breathing-difficulty (reference: no)	1.29 (0.52–3.2)	0.59			0.87 (0.21–3.63)	0.852		
Cough ≥2 weeks + contact history (reference: no)	2.13 (0.91–4.97)	0.082			0.65 (0.16–2.69)	0.557		
Fever ≥2 weeks + weight loss (reference: no)	1.41 (0.9–2.2)	0.131			0.84 (0.58–1.2)	0.342		
Fever ≥2 weeks + fatigue (reference: no)	1.19 (0.16–8.97)	0.869			1.37 (0.32–5.78)	0.673		
Fever ≥2 weeks + breathing-difficult (reference: no)	5.44 (1.13–26.29)	0.035*	5.49 (1.14–26.58)	0.034*				
Fever ≥2 weeks + contact history (reference: no)	0.56 (0.08–4.08)	0.564			0.63 (0.09–4.65)	0.651		
Weight loss + fatigue (reference: no)	0.56 (0.08–4.08)	0.564			0.93 (0.29–2.98)	0.902		
Cough ≥2 weeks + fever ≥2 weeks + weight loss (reference: no)	0.87 (0.68–1.11)	0.257			1.04 (0.85–1.27)	0.692		
Fever ≥2 weeks + weight loss + fatigue (reference: no)	1.51 (0.76–3)	0.24			1.25 (0.74–2.09)	0.401		
Fever ≥2 weeks + weight loss + fatigue + breathing difficulty (reference: no)					3.42 (0.99–11.86)	0.052		
Fever ≥2 weeks + weight loss + fatigue + contact history (reference: no)	2.93 (0.66–13.02)	0.158			3.18 (1.22–8.29)	0.018*	3.05 (1.17–7.98)	0.023*

*Significance at P < 0.05.

PTB = pulmonary TB; cOR = crude odds ratio; CI = confidence interval; aOR = adjusted OR.

Reference:

1. Rahman SM, Maliha UT, Ahmed S, Kabir S, Khatun R, Shah JA, et al. Evaluation of Xpert MTB/RIF assay for detection of Mycobacterium tuberculosis in stool samples of adults with pulmonary tuberculosis. PloS one. 2018;13(9):e0203063.
2. Stop TB Partnership. TECHNICAL INFORMATION NOTE XPERT®MTB/RIF AND ULTRA. 2019.
3. Organization WH. WHO Meeting Report of a Technical Expert Consultation: Non-inferiority analysis of Xpert MTB/RIF Ultra compared to Xpert MTB/RIF. 2017.
